# Optimizing Fuel Consumption Prediction Model Without an On-Board Diagnostic System in Deep Learning Frameworks

**DOI:** 10.3390/s25227031

**Published:** 2025-11-18

**Authors:** Rıdvan Keskin, Egemen Belge, Senol Hakan Kutoglu

**Affiliations:** 1 Department of Electrical Electronics Engineering, Zonguldak Bulent Ecevit University, Zonguldak 67100, Türkiye; egemenbelge@beun.edu.tr; 2 Department of Geomatics Engineering, Zonguldak Bulent Ecevit University, Zonguldak 67100, Türkiye; shakan.kutoglu@beun.edu.tr

**Keywords:** fuel consumption prediction, deep learning, machine learning, long short-term memory, Bayesian optimization

## Abstract

Real-time prediction of the instantaneous fuel consumption rate (FCR) of any vehicle is the key to improving energy efficiency and reducing emissions. The conventional prediction methods, which include an on-board diagnostic (OBD) system, require the specific vehicle parameters and environmental conditions such as air density. We propose a data-driven Bayesian optimization and Monte Carlo (MC) Dropout methods-based long short-term memory (BMC-LSTM) network FCR prediction model using only the vehicle’s throttle position, velocity, and acceleration data. The cost-effective LSTM network-based solution enhances the high-resolution prediction accuracy within a deep learning framework. The network is integrated with the Bayesian optimization and MC-Dropout methods to ensure a probabilistically optimal hyperparameter set and robust networks. The proposed method presents an FCR model that provides calibrated predictions and reliability against distribution drift by probabilistically tuning hyperparameters with Bayesian optimization and quantifying epistemic uncertainty with the MC-Dropout. Our approach requires only vehicle speed, longitudinal acceleration, and throttle position at inference time. Note, however, that the reference FCR used to train and validate the models was obtained from OBD during data acquisition. The performance of the proposed method is compared with a conventional LSTM and Bidirectional LSTM-based multidimensional models, XGBoost and support vector regression-based models, and first- and fourth-order polynomials, which are derived using the least-squares method. The prediction performance of the method is evaluated using Mean Squared Error, Root Mean Squared Error, Mean Absolute, and R-squared statistical metrics. The proposed method achieves a superior $R^{2}$ score and substantially reduces the conventional error metrics.

## 1. Introduction

The instantaneous fuel consumption (FC) of an internal combustion engine-powered vehicle is a metric that represents the fuel economy and the vehicle’s emissions to achieve clean, economic, and sustainable city environments [1,2,3]. Fuel consumption is traditionally measured with an on-board diagnostic (OBD) system; however, some buses, motorcycles, and heavy vehicles are not included [4,5]. Such a system offers the measurement of vehicle parameter IDs, i.e., instantaneous engine revolution, fuel consumption, velocity, temperature, and acceleration, etc. The system can send on-road and off-road parameter IDs to a user device in a wireless environment [6,7,8]. The system, however, requires multiple sensors, which may cause high cost and unreliability, to render the online measurement available in long-term vehicle operation [9]. The key factors affecting the fuel consumption and emission of the vehicles are summarized [10,11,12].

The instantaneous FC of the vehicle is conventionally modeled based on vehicle-specific power (VSP) [13,14]. This method requires multiple specific parameters of a vehicle and the air, i.e., vehicle mass, the translational mass of the rotating components, road grade, coefficient of rolling resistance, ambient air density, headwind into the vehicle, etc., which makes the prediction specific to the vehicle [15]. A one-dimensional polynomial was proposed to estimate the instantaneous power demand of the vehicle using only the vehicle’s specific parameters [16]. One-dimensional motorcycle and bicycle polynomials were constructed to characterize energy consumption rates based on on-road data in Lisbon, Portugal [17]. However, the VSP-based methods evaluate the FC rate of a vehicle using discrete vehicle velocity, which reduces the prediction of the real FC rate in on-road and off-road conditions. In particular, the methods assume that the FC rate is constant during standstill, which can be unrealistic in traffic conditions [18]. A one-dimensional fuel consumption model was proposed using idling energy consumption, in which the energy depends on the movement, the acceleration, the deceleration, and the energy use of the total distance [19]. A one-dimensional polynomial was constructed to represent the fuel consumption and gas emission rates of a vehicle [20,21]. With the emergence of the big data era, a substantial transformation in the field of computational technology has been precipitated. This era enables multidimensional and multi-layer nonlinear mapping to predict the velocity, fuel consumption, and emission characterization of a vehicle [22]. A support vector regression- or artificial neural network (ANN)-based FC rate prediction method was proposed using multiple vehicles’ on-road data [23,24,25]. A standard feed-forward ANN does not address vanishing gradients and struggles with long-range temporal learning. A backpropagation neural network was utilized for the prediction of the FC rate, where a data augmentation method was proposed to obtain a high-precision FC rate [22]. A recurrent neural network (RNN)-based FC rate prediction method was proposed using smartphone measurements [26]. The method results could be insufficient owing to the disappearing and expanding gradient issues encountered during backpropagation, hindering the effective learning of long-term dependencies. In tasks necessitating long-horizon temporal modeling, the absence of gating mechanisms in conventional RNNs results in a substantial degradation due to the limited memory capacity. A comparative study of machine learning methods-based vehicle fuel efficiency prediction methods was proposed to obtain high prediction accuracy in capturing nonlinear mapping [27]. Influencing factors of fuel consumption in heavy-duty trucks were investigated [28,29]. The work collected the data from simulated/lab data, which did not incorporate dynamic factors such as driving behavior and traffic conditions. CatBoost-based fuel consumption rate prediction was proposed for heavy-duty diesel trucks [30]. A long short-term memory (LSTM) network-based fuel consumption prediction method was proposed [31], where the prediction was constructed using Electronic Control Unit (ECU) and simulation-based data. The traffic conditions were not included in the evaluation of the method’s performance. An artificial rabbit optimization algorithm was proposed to optimize the hyperparameters of the LSTM network, i.e., the number of hidden layers, learning rate, and learning decay rate [32]. There have been some works on optimizing the hyperparameters of the RNN networks based on metaheuristic methods [33,34]. The methods may be time-consuming, although such methods do not increase the robustness of the training phase against the network uncertainties. Grid search and random sampling methods are proposed to tune the parameters of the networks [35]. However, these methods may be insufficient for high-dimensional systems. In summary, FCR prediction models require real-time measurements of vehicle parameter IDs from the OBD system to predict the fuel consumption of a vehicle with high precision. The methods that predict the fuel consumption using vehicle velocity, orientation, and acceleration data without requiring an OBD system ignore traffic conditions. As far as we know, no prior work predicts the vehicle FCR in heavy traffic (stop-and-go) and on rough terrain without OBD.

In this context, we propose a parameter-optimized robust LSTM-based fuel consumption model. We have integrated the method with the Monte Carlo (MC) Dropout method to increase the robustness against network uncertainties. We collect real-world driving data from two gasoline vehicles across rough terrain, stop-and-go traffic, and free-flow conditions, yielding a high-quality fuel-consumption dataset. The reference FCR data were obtained from the OBD during the data acquisition process. The proposed method is compared with the FCR prediction models, which are constructed utilizing XGBoost and support vector regression (SVR) methods, a conventional LSTM network, and a least-squares method based on first- and fourth-order polynomials. The contributions of the paper are as follows:

- We propose a method that combines Bayesian optimization with the LSTM network to optimize probabilistically the hyperparameters of the network, i.e., the learning rate, learning rate decay, batch size, the number of hidden layers, and the number of nodes in each hidden layer. The method models the outcome of each hyperparameter combination as a probabilistic function.
- The method presents an FCR prediction model trained using the MC-Dropout method to quantify epistemic uncertainty, improving robustness to distribution shift.
- The method requires only the vehicle translational speed, longitudinal acceleration, and throttle position at inference time.

The remainder of this paper is structured as follows: Section 2 presents the data logger process of the method on the two different vehicles. Section 3 introduces the LSTM network to construct the prediction model. Section 4 presents a summary of the data collection process. Section 5 presents the data-driven FCR model’s performance results using statistical metrics. Section 6 concludes the paper.

## 2. System Description and Problem Formulation

**Figure 1:**
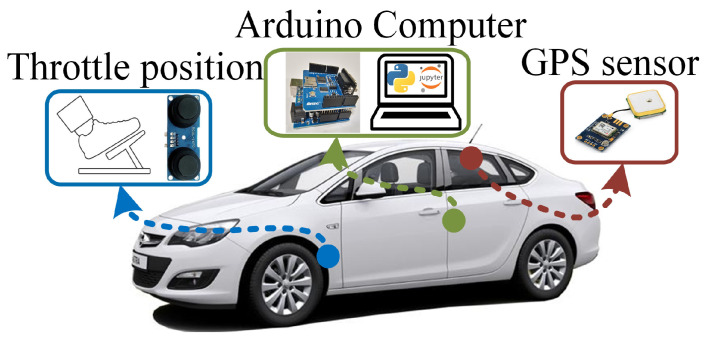
Experimental setup of the proposed fuel consumption rate prediction methodology.

The OBD system gives the instantaneous fuel consumption rate in gasoline- and diesel-powered vehicles. The system gives the parameter IDs based on the specific vehicle. Access to parameter IDs may not be available for certain vehicle types: motorcycles and heavy-duty vehicles. In addition, the OBD-dependent system may not be available in electric vehicles. The fuel flow rate of the engine is given as

(1) $$FCR=\frac{360{\dot{m}}_{air}}{\lambda Bv},$$

where FCR is the fuel consumption rate (g/100km), ${\dot{m}}_{air}$ is the air mass (g/s) obtained from a mass air flow sensor, λ is the actual air/fuel ratio, *B* is the stoichiometric AFR, and *v* is the translational velocity of the vehicle (km/h). The real FCR data, which are obtained from (1), are obtained via the OBD system. The proposed data-driven FCR model is presented in Figure 1, where the system is integrated on two different vehicles: 2018 Opel Astra edition plus and 2014 Nissan Juke. The normalized throttle position (*r*) is obtained using an ultrasonic distance sensor ($r\in [r_{int},1]$), where $r_{int}$ is the initial throttle position including the stationary operation of the vehicle. The data capture stop-and-go behavior and rough-terrain operation. The RCWL-1670 ultrasonic sensor is preferred to measure the throttle/pedal position of the vehicles, since it has high measurement accuracy and stability, capable of measuring distances between 2 cm and 400 cm. It offers high precision of up to ±1 cm beyond the 2 cm blind spot. When calibrating the sensor’s sensitivity, data obtained at known distances can be adjusted using linear regression or software-based correction coefficients. The norm of the vehicle’s position vector, $p={\left[p_{x}\;p_{y}\;p_{z}\right]}^{⊤}$, is given as

(2) $${∥p∥}_{2}=\sqrt{p_{x}^{2}+p_{y}^{2}+p_{z}^{2}},$$

where x,y,z are the Cartesian coordinates. The position data are obtained using the NEO-6M global positioning system (GPS) sensor via an Arduino microprocessor and sent to a host computer to process the data in the Python 3.7 environment. The translational velocity v and acceleration a are obtained by differentiating position along time.

**Figure 2:**
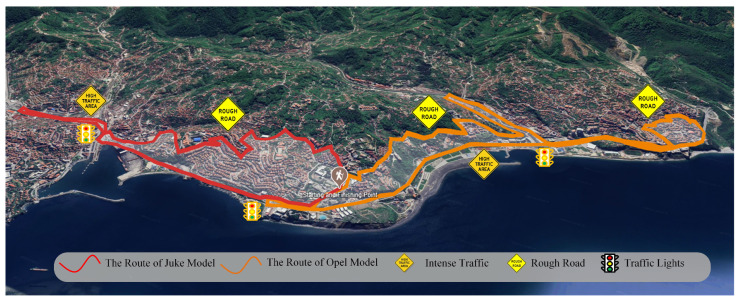
The area that covers the trajectories of the test vehicles.

**Figure 3:**
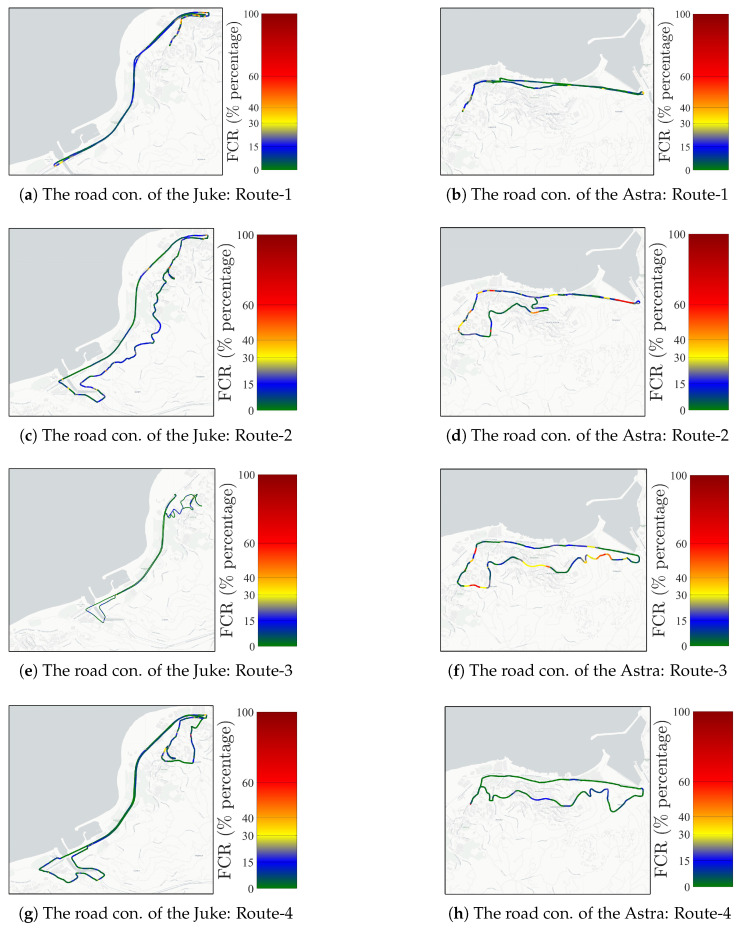

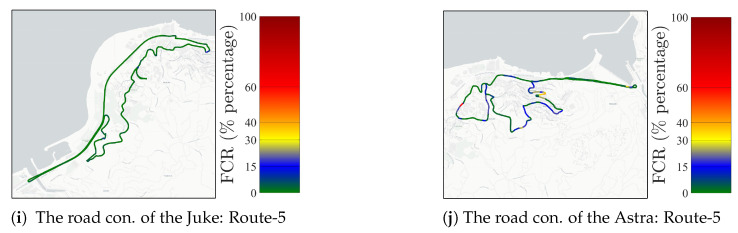
Spatial distribution of the fuel consumption rate of the test vehicles in various trajectory scenarios in 2D space.

**Figure 4:**
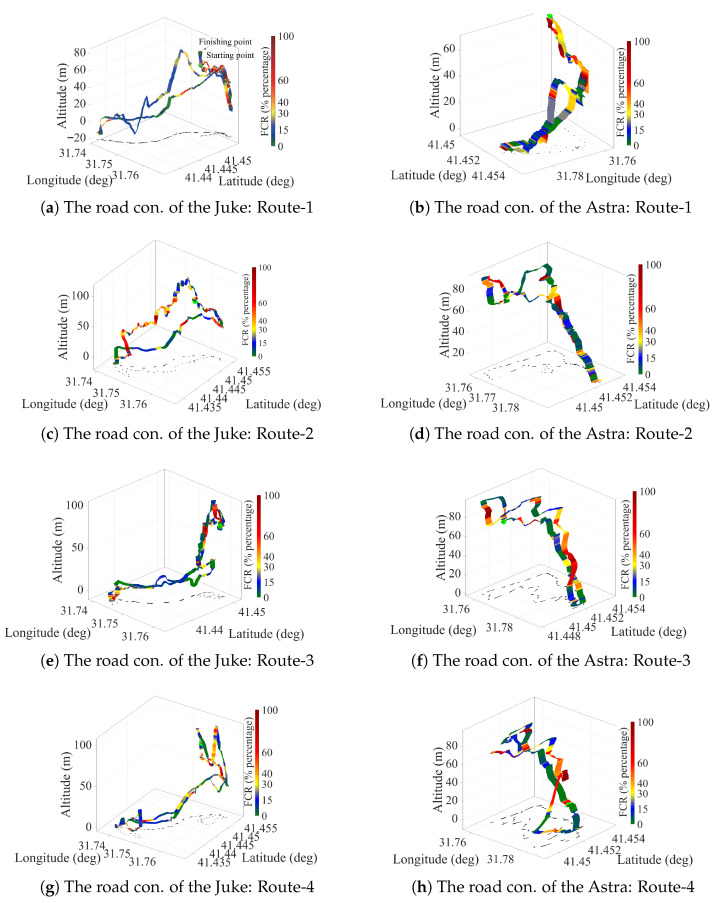

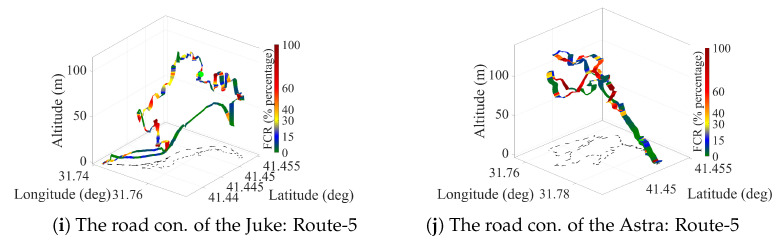
Spatial distribution of fuel consumption rate of the test vehicles in various trajectory scenarios in 3D space; the road conditions of the vehicles (conditions = con.).

We define twenty (20) closed-loop scenarios to consider the traffic conditions: the roads have uphill and downhill parts, and there are road behaviors where pedestrians are more dense or less dense for every vehicle. The targeted area of the trajectories is presented in Figure 2, where the traffic lights, heavy traffic, and road conditions are included. The fuel consumption rates of test vehicles for half of the closed-loop trajectories are presented in Figure 3. Vehicle fuel consumption rates are presented as percentages in the adjacent bar. Green lines represent minimum fuel consumption, while red lines represent maximum fuel consumption. The fuel consumption rates of the test vehicles in three-dimensional space are presented in Figure 4. Lower fuel consumption is naturally expected on flat routes and higher fuel consumption rates on slopes and rough roads, because the traction demand increases, and the engine generates more power when climbing. However, the internal dynamics of the powertrain and the driving strategy can reverse this general trend. The engine’s ability to stay in the efficient zone on the specific fuel consumption map is closely related to the appropriate gear selection, constant acceleration at low but torque-favorable speeds, avoiding unnecessary throttle–brake transitions, and smooth traffic conditions. As a result, the vehicle’s internal dynamics (engine efficiency zone, gear levels, transmission efficiency, and internal losses), the driver’s throttle-to-gear strategy, and the continuity of the traffic flow can override the expected effect of the topography, leading to lower fuel consumption even on rough tracks than on a flat road. Therefore, we propose a deep learning method-based model to predict the instantaneous fuel consumption rate of the test vehicles.

We have constructed three different learning scenarios: (i) Scenario 1: the learning process, which is based on the leave-one-route-out principle, (ii) the learning process, which is based on the train-on-vehicle-A/test-on-vehicle-B principle, (iii) train-on-vehicle-B/test-on-vehicle-A, where vehicle A is the Nissan/Juke model, and vehicle B is the Opel/Astra model, to obtain a leakage-safe learning process.

## 3. Deep Learning-Based Prediction Model

**Figure 5:**
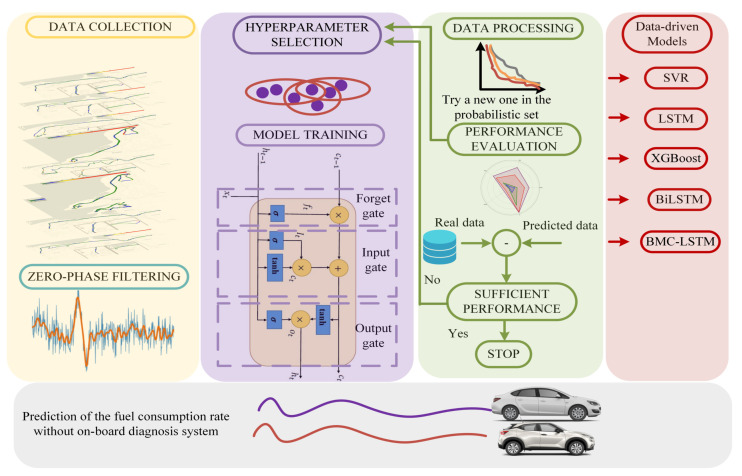
Flowchart representation of the proposed data-driven fuel consumption rate prediction methodology.

This section introduces the proposed bidirectional LSTM network and ridge regression method-based prediction model construction. We construct first- and fourth-order one-dimensional ridge-regularized polynomials as a combination of the vehicle’s translational velocity, acceleration, and the throttle’s position to compare the performance of the proposed method. The flowchart of the proposed data-driven fuel consumption rate predic- tion methodology is presented in Figure 5.

### 3.1. Long Short-Term Memory

**Figure 6:**
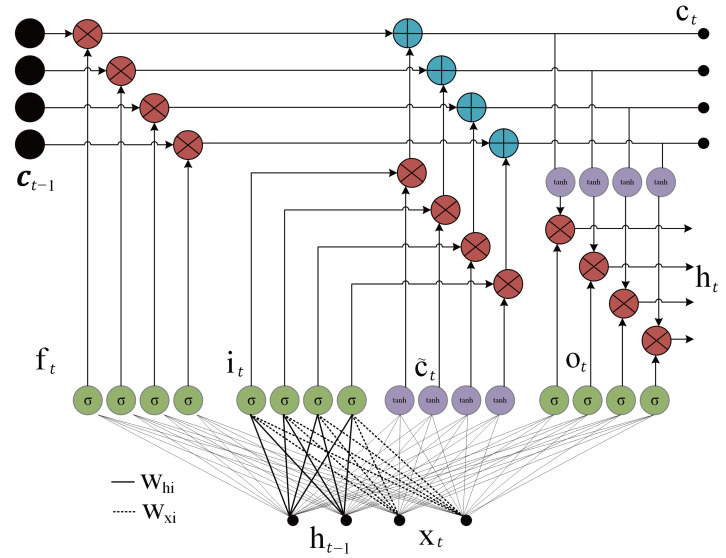
The full structure of the LSTM gates, where the four NN layers are placed in parallel.

RNNs can learn short-term dependencies in the input vectors of a deep learning structure [36]. However, they may cause some gradient problems in the loss function when long-term dependencies are present. The weights of the networks cannot be defined or updated, when there is a dramatic growth in the gradient, which significantly limits the performance of RNN networks, especially in problems involving long-term dependencies. The LSTM networks introduced a solution to this undefined gradient problem in the backpropagation algorithm, since the LSTM has an internal iteration technique without using a nonlinear function. The network is included in the framework of the RNN network. The full structure of an LSTM cell is presented in Figure 6, where *t* defines the current index of the structure, $c_{t}$ is the internal state, $c_{t-1}$ is the previous internal state, $h_{t-1}$ is the previous hidden state, $x_{t}$ is the input pattern, $f_{t}$ is the forgetting gate, $i_{t}$ is the input gate, $o_{t}$ is the output gate, ${\hat{c}}_{t}$ is the input node, $W_{hi}$ is the weight from the hidden gate to the input, and $W_{xi}$ is the weight from the input pattern to the input gate. The LSTM cell structure consists of three gates: the forgetting, input, and output gates, which are defined as

(3) $$f_{t}=\sigma \left(W_{fx}x_{t}+W_{hf}h_{t-1}+b_{f}\right),$$

(4) $$i_{t}=\sigma \left(W_{ix}x_{t}+W_{hi}h_{t-1}+b_{i}\right),$$

(5) $$o_{t}=\sigma \left(W_{ox}x_{t}+W_{ho}h_{t-1}+b_{o}\right),$$

where σ(.) is the sigmoid activation function, $W_{fx}$ is the weight vector from the forgetting gate to the input pattern, $W_{hf}$ is the weight from the hidden state to the forgetting gate, $W_{ox}$ is the weight from output gate to the input pattern, and $W_{ho}$ is the weight from the hidden state to the output gate [37]. The terms $b_{f}$, $b_{i}$, and $b_{o}$ are the bias terms of the relative gates. The sigmoid function constrains the outputs of each gate within [0, 1] in the LSTM cell structure. The forget gate determines which information is discarded from the cell state. The input gate specifies which new information is added to the cell state. The output gate controls which information is transferred to the cell output based on the current cell state. The input node is given as

(6) $${\tilde{c}}_{t}=\tanh\left(W_{cx}x_{t}+W_{hc}h_{t-1}+b_{c}\right),$$

which is a function of the previous hidden state and input. The term tanh(.) is the hyperbolic tangent function. The forgetting and update mechanism of the memory cell of the LSTM network is defined as

(7) $$c_{t}=f_{t}⊙c_{t-1}+i_{t}⊙{\tilde{c}}_{t},$$

where the term ⊙ denotes element-wise multiplication. The differentiation of (7) is given as

(8) $$\frac{\partial c_{t}}{\partial c_{t-1}}=\frac{\partial }{\partial c_{t-1}}\left(f_{t}⊙c_{t-1}+i_{t}⊙{\tilde{c}}_{t}\right),$$

which yields

(9) $$\frac{\partial c_{t}}{\partial c_{t-1}}=\mathrm{diag}\left(f_{t}\right).$$

Therefore, the network can prevent the vanishing problem in the RNN networks. The final main component of the LSTM cell is the output gate, which determines the cell output [38]. This output gate controls which information within the cell is transferred to the outside. The hidden state of the LSTM cell is given as

(10) $$h_{t}=o_{t}⊙\tanh\left(c_{t}\right).$$

### 3.2. Bidirectional LSTM

Bidirectional long short-term memory (BiLSTM), with sequences in both forward and backward directions, is an extension of the classical LSTM to exploit past and future context [39]. Standard LSTMs achieve strong performance on sequence modeling tasks. This LSTM network is capable of learning long-term dependencies in data. However, during the learning process of the network, training is performed only on the information from the past. The BiLSTM deep learning network could complete the learning process by training data from the past and the future simultaneously. The BiLSTM deep learning structure feeds the input data simultaneously to two separate LSTM networks. While one LSTM network processes the input data in the forward direction, the other LSTM network could process the same data in the reverse direction. As a result, the comprehensive learning process is performed in both forward and backward directions at each time step. The forward hidden state in BiLSTM is given as

(11) $${\vec{h}}_{t}=\sigma \left(W_{h\vec{x}}x_{t}+W_{\vec{h}\vec{h}}\;{\vec{h}}_{t-1}+b_{\vec{h}}\right),$$

where $W_{h\vec{x}}$ and $W_{\vec{h}\vec{h}}$ are the forward weight matrices, and $b_{\vec{h}}$ is the forward bias vector. The backward hidden state in BiLSTM is defined as

(12) $${\overset{\leftarrow }{h}}_{t}=\sigma \left(W_{h\overset{\leftarrow }{x}}x_{t}+W_{\overset{\leftarrow }{h}\overset{\leftarrow }{h}}\;{\overset{\leftarrow }{h}}_{t+1}+b_{\overset{\leftarrow }{h}}\right),$$

where $W_{h\overset{\leftarrow }{x}}$ and $W_{\overset{\leftarrow }{h}\overset{\leftarrow }{h}}$ are the backward weight vectors, and $b_{\overset{\leftarrow }{h}}$ is the backward bias vector [40]. Therefore, the forward and backward hidden layers in BiLSTM are evaluated to increase the prediction performance. The output of the network is given as

(13) $$y_{t}=W_{\vec{y}h}{\vec{h}}_{t}+W_{\overset{\leftarrow }{y}h}{\overset{\leftarrow }{h}}_{t}+b_{y},$$

where $W_{\vec{y}h}$ and $W_{\overset{\leftarrow }{y}h}$ are weights of the output layer from the forward and backward hidden layers, and $b_{y}$ is the bias term of the network [41].

### 3.3. Bayesian Method-Based Hyperparameter Optimization

In RNN methods, the hyperparameters of the network are typically fixed and adjusted manually based on the designer’s prior experience. There are some methods to optimize these parameters using metaheuristic optimization methods, i.e., gray wolf, particle swarm, genetic algorithm, etc. [26]. However, such an approach dramatically increases the time of the training process. Such methods can be slow and do not address model uncertainty. There are basic methods to optimize the parameters in a lower time-consuming context: grid search and random sampling. The Bayesian method treats the verification error as a black-box cost and models this function probabilistically with a Gaussian process. In each iteration, a hyperparameter vector is chosen to train the model, and a probabilistic surrogate model is fitted. The posterior, which is a distribution defined by the Bayesian method, is given by

(14) $$\rho \left(\vartheta |D\right)=\frac{\rho (D|\vartheta )\rho (\vartheta )}{\rho (D)},$$

where

(15) $$\begin{array}{cc} \; & \rho (D)=\int \rho (D|\vartheta )\rho (\vartheta )d\vartheta , \end{array}$$

where D is the input-output dataset, ϑ is the the layer parameter space W∈ϑ, and ρ(·) is the distribution. The predictive posterior distribution of the next desired output $y_{k+1}$ is given by

(16) $$\rho \left(y_{k+1}|x_{k+1},D\right)=\int \rho \left(y_{k+1}|x_{k+1},\vartheta \right)\rho \left(\vartheta |D\right)d\vartheta ,$$

where *k* is the index of samples. The posterior is updated to determine the next hyperparameter vector. The method utilizes posterior uncertainty to select new evaluation points and approach the global optimum with minimal attempts. For a given set of hyperparameters, θ, the optimal set is given by

(17) $$\theta_{opt}=\arg\max_{\theta }\;log\;\rho \left(D,\theta \right),$$

where $\theta_{opt}$ is the optimal hyperparameter set [38]. Dropout entails the exclusion of random groups of graph nodes during training, with a new random subset selected for each forward pass in graph neural networks [42]. MC-dropout (MC) approximately marginalizes the weighted sequence by keeping dropout enabled in the test on this chosen structure; therefore, generalization and calibration are improved due to epistemic uncertainty [43]. The approximate posterior function of the MC-Dropout is given by

(18) $$q\left(\vartheta \right)=\sum_{1\leq k\leq L}\sum_{z_{k}=0,1}\delta_{{\hat{\vartheta }}_{1}z_{1},\ldots ,{\hat{\vartheta }}_{L}z_{L}}\left(\vartheta_{1},\ldots ,\vartheta_{L}\right)\cdot \underset{q(z)}{\underbrace{\rho^{\sum z_{k}}{\left(1-\rho \right)}^{\sum 1-z_{k}}}},$$

where $\hat{\vartheta }$ represents the learned weights, *L* is the dimension of the parameter vector, $\delta_{c}\left(\vartheta \right)$ is the point mass at *c*, and *z* is the Bernoulli dropout mask [44]. Substituting p(ϑ,X,Y) of (16) with (18), the closed form of the MC-Dropout predictive posterior is given by

(19) $$p_{MC}\left(y_{k+1}|x_{k+1},D\right)=\sum_{z\in {\left[0,1\right]}^{L}}\rho \left(y_{k+1}|x_{k+1},\hat{\vartheta }⊙z\right)\cdot q\left(z\right).$$

### 3.4. Ridge-Regularized Polynomial Model

A feature input vector is chosen as a combination of the velocity, acceleration, throttle position, and the time derivative of the throttle position $x_{k}={\left[\;v_{k}\;a_{k}\;r_{k}\;{\dot{r}}_{k}\;{\left(va\right)}_{k}\;\right]}^{⊤}$, where k=1,...,N is the data point. A predicted ridge polynomial is given by

(20) $${\hat{y}}_{k}=\beta_{0}+\sum_{j=1}^{m}\beta_{j}\;\phi_{j}\left(x_{k}\right),$$

where *m* is the number of features, *j* is the feature index, ${\left\{\phi_{j}\left(\cdot \right)\right\}}_{j=1}^{m}$ are polynomial features, $\beta_{0}$ is the bias term of the polynomial, and β is the coefficient of the polynomial $\beta ={\left[\beta_{1},\ldots ,\beta_{m}\right]}^{⊤}$. The loss function of the ridge polynomial is defined by

(21) $$J\left(\beta_{0},\beta \right)=\sum_{k=1}^{N}({y_{k}-\beta_{0}-\sum_{j=1}^{m}\beta_{j}\phi_{j}\left(x_{k}\right)}^{2})+\gamma \sum_{j=1}^{m}\beta_{j}^{2},$$

where *N* is the number of samples, $y_{k}$ is the *k*-th real value, and γ is a non-dimensional positive coefficient. The optimal coefficients of the polynomial are given by

(22) $$\left(\beta_{0}^{⋆},\;\beta^{⋆}\right)=\arg\min_{\beta_{0},\;\beta }\;J\left(\beta_{0},\beta \right).$$

## 4. Data Collection

In this study, data were collected as a time series with a 1 s sampling time to propose a deep learning-based vehicle fuel consumption estimation model. The sensor data, including the fuel consumption rate, GPS positions, and vehicle accelerator throttle/pedal position, are fused on the Arduino Uno card to be streamed to the host computer via serial communication interface at a band rate of 230,400. Thus, since all streams were time-stamped with the same Arduino time base, channel alignment is assumed to be achieved within the 1 Hz common sampling grid; no additional delay model was used. The internal sampling rates of the sensors and the internal interface delays were not measured separately in this study; the decisive end-to-end sampling rate used in the experimental setup was 1 Hz. This dataset, which consists of *N* = 23,701 samples, is processed using a low-pass filter. We have designed a second-order discrete-time Butterworth filter which has a 0.12 cut-off frequency. The filter order is chosen as two to avoid undesired signal delays. The filter is applied on a Python 3.7 in Jupyter Notebook. environment using the filtfilt command. For the filtering part of the experimental setup, we did not filter the signals during the real-time operation (the filtfilt command can not be used in real-time operation), Therefore, we have filtered the dataset in the offline data processing part of the study. The filter is given by

(23) $$H\left(z\right)=\frac{0.09z^{2}+0.18z-0.0913149}{z^{2}-0.98240579z+0.34766539}.$$

In real-time operation, the Butterworth filter can be implemented in causal processors without the zero-phase advantage, since the operating/resampling frequency is chosen as the sampling frequency of the serial port. This process adds some delays to the dataset; however, the additional delay of the filtering process is assumed to be under the acceptable tolerance limit, since the filter is applied to the whole dataset. The networks are trained using the dataset to predict the model using the Python Keras 1.4.7 and TensorFlow 2.13.0 Libraries. The data information of the acquired dataset, which is presented in Figure 3 and Figure 4, is given in Table 1.

## 5. Performance Results of the Data-Driven Prediction Model

This section presents the performance results of the prediction method to evaluate the effectiveness of the proposed methodology. The statistical performance metrics are given as

(24) $$\mathrm{RMSE}=\sqrt{\frac{1}{N}\sum_{k=1}^{N}{\left(y_{k}-{\hat{y}}_{k}\right)}^{2}},$$

(25) $$\mathrm{MSE}=\frac{1}{N}\sum_{k=1}^{N}{\left(y_{k}-{\hat{y}}_{k}\right)}^{2},$$

(26) $$\mathrm{MAE}=\frac{1}{N}\sum_{k=1}^{N}\left|y_{k}-{\hat{y}}_{k}\right|,$$

(27) $$R^{2}=1-\frac{\sum_{k=1}^{N}{\left(y_{k}-{\hat{y}}_{k}\right)}^{2}}{\sum_{k=1}^{N}{\left(y_{k}-\bar{y}\right)}^{2}},$$

(28) $$\mathrm{Loss}=\frac{1}{N}\sum_{k=1}^{N}\log\left(\cosh\left(y_{k}-{\hat{y}}_{k}\right)\right),$$

where ${\hat{y}}_{k}$ is the *k* -th predicted value of the model, $\bar{y}$ is the mean of the real values, cosh(x) is the hyperbolic cosine function, MSE is the mean square error, MAE is the mean absolute error, $R^{2}$ is the coefficient of determination, Loss is the loss function of the deep learning methods, and RMSE is the root mean square error. Table 2 presents the capacity and regularization regimes of the three deep learning methods. For BiLSTM and LSTM, a deterministic and medium-capacity setup is adopted with fixed 128–128 units in the two hidden layers, zero dropout, and learning rate $10^{-3}$ (total number of layers 4, epoch 60, batch size 16 fixed). In contrast, in BMC-LSTM, the first and second hidden layer value ranges are defined as [64,180], dropout [0.1,0.5], and learning rate $\left[10^{-4},10^{-2}\right]$, thus adapting the model complexity, regularization power, and optimization speed together. This design allows BMC-LSTM to adjust the bias–variance balance according to the scenario and data distribution, control the risk of overfitting (dropout), and keep the training dynamics (learning rate) in a stable region, while fixing the epoch and batch size ensures a fair comparison between different architectures.

For the parameters of the XGBoost method, the learning rate is chosen as 0.05, the number of estimators is chosen as 300, and the number of states is chosen as 42. For the parameters of the SVR method, the regularization coefficient is chosen as 100, the gamma coefficient is chosen as 0.1, epsilon is chosen as 0.1, and the kernel of the SVR model is defined as a radial basis function.

**Figure 7:**
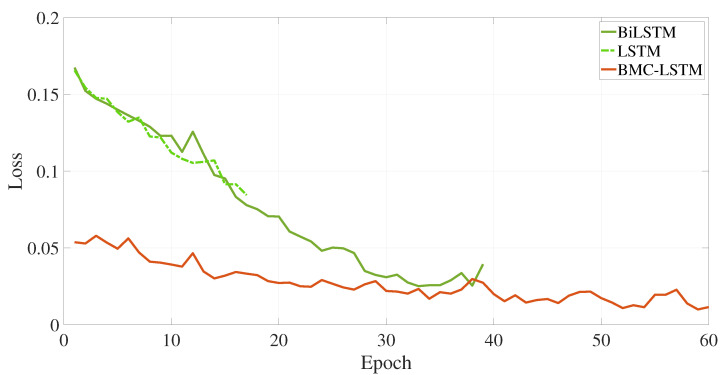
The training loss performance of the deep learning-based methods.

**Figure 8:**
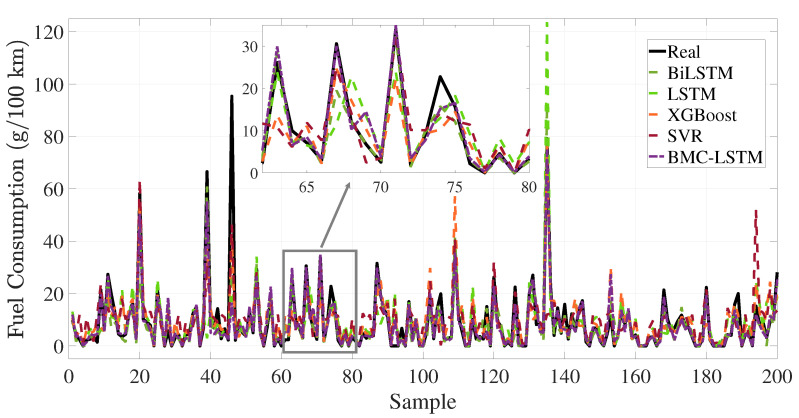
The prediction results on the sample data of the deep learning- and the machine learning-based methods.

**Figure 9:**
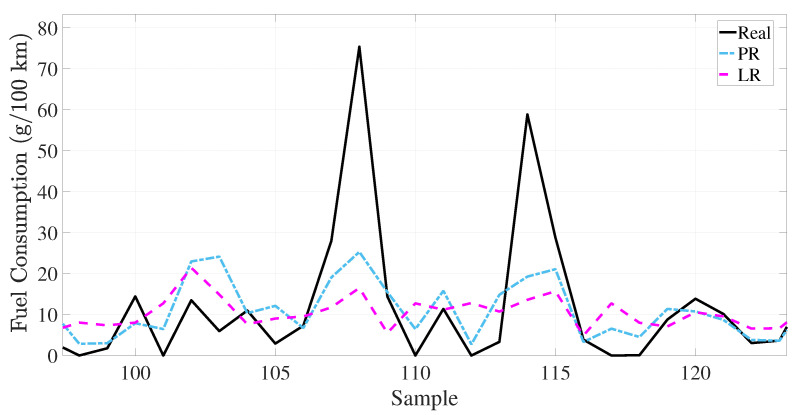
The prediction results on the sample data of the linear regression-based polynomials.

**Figure 10:**
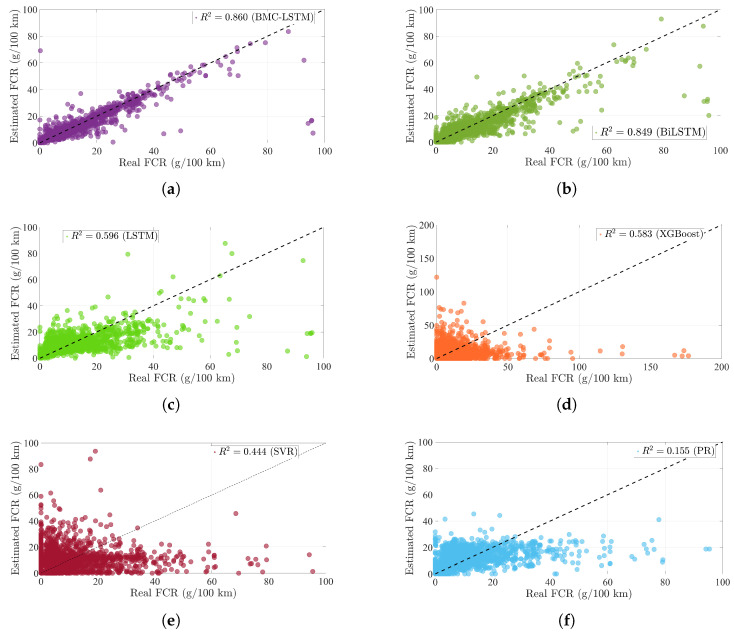
The scatter metric performance of the methods: (**a**) $R^{2}$ performance of the proposed method, (**b**) $R^{2}$ performance of the BiLSTM method, (**c**) $R^{2}$ performance of the LSTM method, (**d**) $R^{2}$ performance of the XGBoost method, (**e**) $R^{2}$ performance of the SVR method, (**f**) $R^{2}$ performance of the PR method.

The training loss performance of the deep learning-based method is presented in Figure 7. The deep learning method is executed in 60 iterations. The proposed deep learning methodology has a minimum loss function value at the 60 *i*-th iteration. The BiLSTM and the proposed deep learning method have the loss value at 38 *i*-th iteration. However, the fluctuation of the loss function is the maximum in the BiLSTM. This situation causes the overfitting problem during the training process. The proposed BMC-LSTM method has a roughly monotonically decreasing learning curve during the training process. Furthermore, the proposed deep learning model presents robustness against network uncertainties and the overfitting problem. The FCR prediction performance of the models is presented in Figure 8 and Figure 9. The prediction performance of the fuel consumption rate in this model is tested on 200 samples. The results indicate that the BMC-LSTM based model has a superior FCR prediction performance. In Figure 9, the PR denotes the fourth-order polynomial ridge-based polynomial, and LR denotes the first-order linear ridge-based polynomial. It is emphasized that the deep learning-based methods indicate more than sufficient FCR prediction performances. The $R^{2}$ scatter performance of the methods is presented in Figure 10. It is stated that the prediction performances of BiLSTM and the proposed deep learning approach are close to each other. However, the prediction performance of the proposed method is slightly better than that of BiLSTM in Scenario 1.

Table 3 shows that BMC–LSTM achieves the lowest error (RMSE/MSE/MAE) and highest agreement ($R^{2}$) values in all three scenarios. Specifically, Scenario 3 shows the best performance by far with RMSE =4.57, MSE =20.89, MAE =2.14, and $R^{2}=0.95$, while Scenario 2 maintains strong results, with RMSE =5.40 and $R^{2}=0.91$. In Scenario 1, BMC–LSTM produces an RMSE close to BiLSTM (5.54 vs. 5.76), while exhibiting a more robust error distribution with a significantly lower MAE (2.04). While the two-layer LSTM is competitive in some cases (e.g., RMSE =4.97, $R^{2}=0.92$ in Scenario 3), BMC–LSTM consistently outperforms all metrics. Classical linear/multinomial regressions (LR/PR) are significantly weaker, while SVR and XGBoost are moderate; these results confirm that deep Bayesian uncertainty modeling provides a significant advantage in terms of both accuracy and stability. The inference times of the BiLSTM, LSTM, SVR, XGBoost, and BMC-LSTM are 39.047 ms/sample, 36.292 ms/sample, 87.97 ms/sample, 1.8 ms/sample, and 38.171 ms/sample, respectively. When we evaluate the performance of these learning models in the context of the requirements of a typical telematics flow, an inference time of 38.171 ms/sample means our model can process 26 samples per second. This rate meets the requirements of the most standard OBD-II, GPS, and ultrasonic distance sensor acquisition rates of 10 Hz (10 samples per second) or 20 Hz (20 samples per second). As a result, although our BMC-LSTM, BiLSTM, and LSTM models have higher inference times than XGBoost, these models remain within the maximum latency limits required by real-time telematics systems, in exchange for their more complex pattern capture and high accuracy. This situation indicates that our BMC-LSTM model achieves both high accuracy and practical applicability.

**Figure 11:**
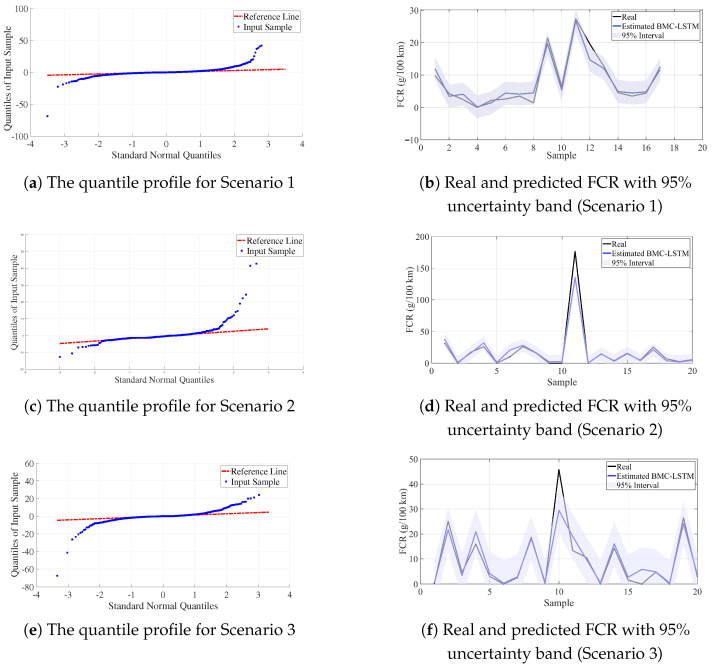
Calibration and prediction views for BMC–LSTM in three scenarios: (**a**,**c**,**e**) residual/distribution fit with standard normal Q–Q diagrams (Red lines are the standard distribution and blue lines are the input samples); (**b**,**d**,**f**) comparison of actual and predicted FCR with 95% uncertainty band.

The three scenarios presented in Table 4 consistently validate the cross-vehicle generalization ability and uncertainty calibration of the BMC-LSTM model. The prediction interval coverage probability (PICP) values (S1: 94.12%, S2: 96.51%, S3: 95.86%) are very close to the nominal 95% coverage target; in particular, the 96.51% value at S2 indicates that the intervals are slightly conservative and well-calibrated, while S1 and S3 show neutral calibration close to the target. The low out-of-distribution (OOD) error rates (S1: 5.88%, S2: 2.96%, S3: 3.89%) indicate stable performance even on ODD samples, while confirming the robustness in the cross-vehicle test (S2: training on Nissan → Astra; S3: training on Astra → Nissan Juke); Furthermore, the lower OOD ratio of S2 compared to S3 suggests a stronger overlap of feature spaces in the Nissan→Astra direction. Although the NLL values (S1: 1.99, S2: 3.10, S3: 2.94) increased to an expected extent in scenarios involving vehicle transfers (moderate difficulty in probabilistic fitting), when considered together with PICP and OOD, the prediction distributions appear to produce a reliable uncertainty signal for practical use. In conclusion, in the trip-by-trip evaluation, BMC-LSTM provides a fuel prediction model with well-calibrated prediction intervals, low out-of-distribution error, and acceptable probabilistic fit in both within-vehicle and cross-vehicle tests. The calibration and prediction curves for the proposed BMC-LSTM method in three scenarios are presented in Figure 11.

**Figure 12:**
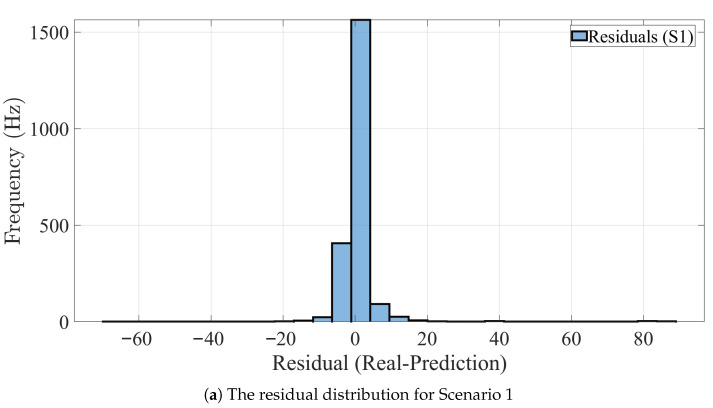

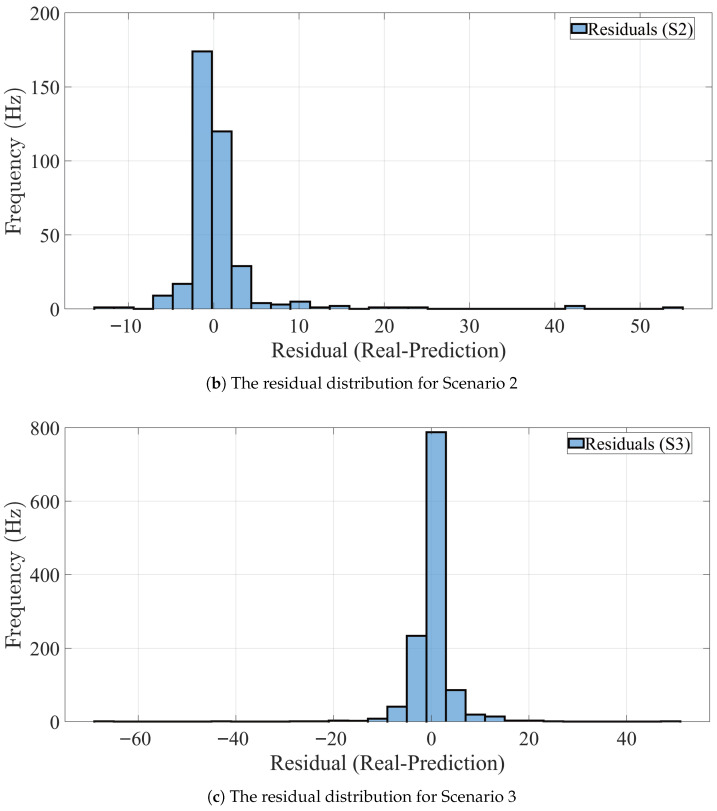
The residual distribution of the BMC-LSTM model in all scenarios.

The residual distribution of the proposed method is presented for scenarios 1–3 in Figure 12. In general, the residual errors are centralized and largely symmetrical around zero, indicating that the model makes reliable and consistent predictions.

The current dataset is limited to two gasoline vehicles (Astra, Juke), predominantly urban routes in a single region, and predominantly mild/dry weather conditions; therefore, vehicle diversity (engine displacement, gear type, age/weight/powertrain architecture), road/topography spectrum (highway, high altitude, long ascents/descendants), driving styles, and seasonality are underrepresented. The 1 Hz sampling may partially under-sample short-term transition regimes (hard throttle/brake, gear shifts); GPS/ultrasonic sensor-induced noise/calibration errors and heteroskedasticity measurement uncertainty may impact data quality. Additionally, systematic testing has not been conducted with different fuel types (diesel/hybrid) and under different climate/terrain conditions; no transfer/adaptive learning framework explicitly designed for transfer to unknown vehicle models has been implemented. These limitations impose inherent limits on the generalization and external validity of the model under dispersion-shift conditions.

## 6. Conclusions

In this paper, we have proposed the Bayesian and MC-Dropout-based LSTM deep learning methodology to predict the fuel consumption rate of vehicles, where the ground-truth FCR data are obtained from OBD in the data acquisition process. Bayesian optimization probabilistically optimized the LSTM’s hyperparameters to find the optimal structure and learning rates. MC-Dropout reduced the model’s epistemic uncertainty, improving the predictive stability and generalization ability of this optimized LSTM. Therefore, the method provides calibrated predictions and improved reliability under distribution shift. The VSP-like first- and fourth-order polynomials are constructed for comparison purposes. The proposed method is compared with advanced learning methods, i.e., XGBoost, SVR, LSTM, and BiLSTM to verify the effectiveness and robustness of the method. Four different performance criteria are selected to analyze the effectiveness of the proposed deep learning methodology. In the context of the $R^{2}$ score, the one-dimensional LR- and PR-based models are insufficient to predict the FCR rate, since such models could achieve prediction performance in all scenarios. The XGBoost, SVR, LSTM, and BiLSTM network-based FCR models achieve 58%, 44%, 60%, and 85% prediction performance in Scenario 1. However, it is observed that the networks may be sensitive to network uncertainties and the overfitting problem, as can be seen from the training loss curve. The BMC-LSTM deep learning model has the minimum RMSE, MSE, and MAE values in all three scenarios. The $R^{2}$ value of the proposed BMC-LSTM deep learning model in Scenarios 1, 2, and 3 has been obtained as 0.86, 0.91, and 0.95, respectively.

## 7. Future Work

We are planning to expand the dataset to include multi-city/multi-climate data, different road classes, and vehicle types (diesel, hybrid). We will focus on Internet of Things (IoT) and mobile application integrations to translate the developed model into practical applications. Future goals include exploring structures based on combining data from phone sensors with sensors other than the OBD system (sensor fusion) and privacy-preserving federated learning infrastructures that allow the model to perform real-time inference directly on edge devices.

## Figures and Tables

**Table 1:** The data info of each test drive presented in Figure 3 and Figure 4 (Dur. = duration, cond. = conditions, S = scenario.

Drive Name	Samples (*N*)	Dur. Time (min)	Road Cond.	Role in the S1/S2/S3
Juke/Route-1	1160	19.3	Flat	Training/Training/Test
Juke/Route-2	1051	17.52	Extra Urban	Training/Training/Test
Juke/Route-3	1018	16.97	Semi-flat	Training/Training/Test
Juke/Route-4	1395	23.25	Flat	Test/Training/Test
Juke/Route-5	1202	20	Urban	Test/Training/Test
Astra/Route-1	714	11.9	Flat	Training/Test/Training
Astra/Route-2	1335	22.25	Semi-Flat	Training/Test/Training
Astra/Route-3	861	14.35	Urban	Training/Test/Training
Astra/Route-4	776	12.93	Urban	Test/Test/Training
Astra/Route-5	1209	20.15	Extra Urban	Test/Test/Training

**Table 2:** The hyperparameters of the deep learning networks.

Model	Hidden Layers (First and Second)	Layers	Dropout	Learning Rate	Epochs	Batch Size
BiLSTM	128–128	4	0	0.001	60	16
LSTM	128–128	4	0	0.001	60	16
BMC-LSTM	[64–180], [64–180]	4	[0.1–0.5]	[0.0001–0.01]	60	16

**Table 3:** The performance comparison of vehicle emission estimation models.

Scenario Number	Model	RMSE	MSE	MAE	$R^{2}$
1	BiLSTM	5.76	33.15	2.92	0.85
	2-Layer LSTM	9.42	88.75	4.94	0.60
	PR	13.77	189.71	7.34	0.15
	LR	14.52	210.75	8.13	0.06
	XGBoost	9.57	91.62	4.82	0.58
	SVR	11.05	122.08	5.91	0.44
	BMC-LSTM	5.54	30.66	2.04	0.86
2	BiLSTM	8.12	65.95	3.65	0.79
	2-Layer LSTM	5.75	33.11	2.37	0.90
	PR	12.67	160.49	5.91	0.09
	LR	12.85	165.15	7.37	0.06
	XGBoost	13.48	181.62	5.89	0.45
	SVR	14.07	198.09	6.61	0.40
	BMC-LSTM	5.40	29.14	2.23	0.91
3	BiLSTM	5.70	32.46	2.69	0.91
	2-Layer LSTM	4.97	24.74	2.55	0.92
	PR	14.01	196.37	7.68	0.22
	LR	15.60	243.30	9.11	0.03
	XGBoost	11.61	134.79	5.17	0.65
	SVR	13.55	183.55	6.46	0.52
	BMC-LSTM	4.57	20.89	2.14	0.95

**Table 4:** The metrics of the proposed emission estimation model.

Metric	Scenario 1	Scenario 2	Scenario 3
PICP	94.12%	96.51%	95.86%
NLL	1.9855	3.1049	2.9385
OOD	5.88%	2.96%	3.89%

## Data Availability

The original data supporting the findings of this study is openly available on GitHub at a permanent repository https://github.com/rkeskin/LSTM-GRU-Fuel-consumption-Vehicle-emission-prediction/issues/1#issue-3625340459 (accessed on 12 November 2025).
